# Explaining regional variation in elective hip and knee arthroplasties in Finland 2010 − 2017—a register-based cohort study

**DOI:** 10.1186/s12913-022-08305-7

**Published:** 2022-07-09

**Authors:** Kristiina Manderbacka, Markku Satokangas, Martti Arffman, Eeva Reissell, Ilmo Keskimäki, Alastair H. Leyland

**Affiliations:** 1 Welfare State Research and Reform Unit, Finnish Institute for Health and Welfare(THL), P.O.Box 30, 00271 Helsinki, Finland; 2 Network of Academic Health Centres and Department of General Practice and Primary Health Care, University of Helsinki, P.O. Box 20, 00014 Helsinki, Finland; 3 Faculty of Social Sciences, University of Tampere, 33014 Tampere, Finland; 4 MRC/CSO Social and Public Health Sciences Unit, University of Glasgow, Glasgow, UK

**Keywords:** Joint arthroplasty, Medical practice variation, Health services research, Register-based study, Equity in health care

## Abstract

**Background:**

A persistent research finding in industrialised countries has been regional variation in medical practices including elective primary hip and knee arthroplasty. The aim of the study was to examine regional variations in elective total hip and knee arthroplasties over time, and the proportions of these variations which can be explained by individual level or area-level differences in need.

**Methods:**

We obtained secondary data from the Care Register for Health Care to study elective primary hip and knee arthroplasties in total Finnish population aged 25 + years between 2010 and 2017. Two-level Poisson regression models – individuals and hospital regions – were used to study regional differences in the incidence of elective hip and knee arthroplasties in two time periods: 2010 − 2013 and 2014 − 2017. The impact of several individual level explanatory factors (age, socioeconomic position, comorbidities) and area-level factors (need and supply of operations) was measured with the proportional change in variance. Predictions of incidence were measured with incidence rate ratios. The relative differences in risk of the procedures in regions were described with median rate ratios.

**Results:**

We found small and over time relatively stable regional variation in hip arthroplasties in Finland, while the variation was larger in knee arthroplasties and decreased during the study period. In 2010 − 2013 individual socioeconomic variables explained 10% of variation in hip and 4% in knee arthroplasties, an effect that did not emerge in 2014 − 2017. The area-level musculoskeletal disorder index reflecting the need for care explained a further 44% of the variation in hip arthroplasties in 2010 − 2013, but only 5% in 2014 − 2017 and respectively 22% and 25% in knee arthroplasties. However, our final models explained the regional differences only partially.

**Conclusions:**

Our results suggest that eligibility criteria in total hip and knee arthroplasty are increasingly consistent between Finnish hospital districts. Factors related to individual level and regional level need both had an important role in explaining regional variations. Further study is needed on the effect of health policy on equity in access to care in these operations.

**Supplementary Information:**

The online version contains supplementary material available at 10.1186/s12913-022-08305-7.

## Background

A persistent research finding in industrialised countries has been regional variation in medical practices. In a recent systematic review, Corallo et al. [1] reported a large number of studies examining medical practice variation from OECD countries covering regional variations in hospital admissions due to a range of chronic conditions as well as elective surgical procedures including total hip and knee arthroplasties. While it is likely that there are regional variations in both prevalence of osteoarthritis and population structure, researchers have suggested that at least part of this variation is unwarranted as within country variation does not vanish when factors related to need for procedures and population sociodemographic structure are controlled for [2]. Unwarranted variation could be due to many reasons, such as regional differences in accessibility of facilities performing these procedures and differences in supply of procedures in relation to regional need.

A number of studies from different countries have focused on hip and/or knee arthroplasties and reported relatively large regional variations for their rates [3–11]. In addition to potential regional variation in medical need and eligibility for treatment including comorbidity, potential factors behind these differences include regional variation in socioeconomic or age-structure of patients, care accessibility [8, 11–14], as well as other need [3, 4, 15] and supply-side factors (e.g. hospital type, availability of orthopaedic specialists and their propensity to operate) [3–5, 8, 9, 15, 16].

The sociodemographic composition of the region may have an effect on regional variation in these operations as there is evidence of an association between ageing of the population, previous heavy physical workload (e.g. work involving heavy lifting or farming) and the occurrence of hip and knee osteoarthrosis [17–20]. In the case of osteoarthrosis, a study from England has estimated that in the early 1990s the incidence of idiopathic osteoarthrosis was 3–11% larger than the annual operation rate [21]. This study examines regional variations in elective total hip and knee arthroplasties over time, and whether these can be explained by individual or area-level differences in need.

## Methods

### Aim of the study

The aim of the current study was to examine (1) trends in regional variation in elective primary hip and knee arthroplasties and to analyse (2) how much of the variation can be explained by individual level factors including gender and age, comorbidities and socioeconomic position of the patients, or area level differences in population need, and (3) whether the effects of these factors have changed over time. Elective primary hip and knee operations are an interesting case for examining the reasons behind variation as there are no clear-cut criteria for need of these operations.

The Finnish health-care system provides a good case for examining variations in joint arthroplasties as the system is universal in coverage and therefore, in general, supports equity in access to health care according to need. Municipalities provide public primary health care for the whole population through health centres. In addition, employers organize mandatory preventive occupational health care for their employees and can also organize curative primary care services. There are also private primary care services available for the population, but user fees can be a barrier to accessing them as private care is only partially reimbursed to patients [22]. The public system is mainly financed by tax revenues and user-fees are generally low. The system supports evidence-based care as there are accepted guidelines for the treatment of altogether 106 conditions including guidelines for treatment of osteoarthrosis of knee and hip in the National Current Care guidelines system [23].

### The study data

We obtained information on patients undergoing hip and knee arthroplasties for the total Finnish population aged ≥ 25 years in 2010 − 2017 from the routinely collected Finnish Care Register for Health Care maintained by the Finnish Institute of Health and Welfare (THL). To guarantee maximum coverage of the procedures we further complemented the hospital discharge register data using the Finnish Arthroplasty Register (FAR) maintained by the THL. Hospitalisations including these procedures from Care Register and FAR were combined if there was a maximum one-day gap separating the periods. A maximum of two primary procedures per person were examined. We formed annual cohorts for all individuals with primary hip or knee arthroplasty according to the NOMESCO Classification of Surgical Procedures [24]. The total Finnish population aged 25 or older was used as the population at risk. In total Finland had about 5.5 million inhabitants during the study period.

The annual FOLK data set maintained by Statistics Finland was used to obtain individual level data for gender, age, municipality of residence, level of education, family net income per consumption unit using the OECD framework [25] and occupational social class for each individual. Level of education was classified as basic, secondary, and higher. Occupational social class was classified as upper white-collar employees, lower white-collar employees, blue-collar workers, farmers and others. For those no longer in the workforce, information of occupation was traced back to previous population censuses. Income was classified into quintiles. A summary measure including five chronic diseases (chronic heart failure, COPD, diabetes, hypertension and dementia) introduced by Saver et al. [26] as an indicator for comorbidity was collected for each year from the diagnoses recorded at Care Register for inpatient hospitalisations and specialist outpatient visits during the preceding five years for the population at risk. We classified these comorbidities as 0, 1 and 2 + comorbidities in the analyses.

As the geographic unit of analyses we used region of residence which was operationalised as hospital districts (run by the regional federations of municipalities), responsible for organising public specialist services in the area [22]. Each municipality must belong to a hospital district. There are currently 20 hospital districts in Finland, with each usually having a single hospital that provides hip and knee arthroplasties. Publicly funded hospitals produce about 95% of all specialised inpatient services in Finland. The municipality of residence was linked for each individual at the end of the year preceding the procedure. The small autonomous region of the Åland Islands was excluded from the analyses. Age at hospitalisation was classified into 5-year age brackets but those aged 25–49 years were merged into one group and those 80 years or older into one group. Those with operation due to hip fracture (ICD-10 code S72.0) as well as those living permanently in institutions were excluded from analyses.

As we did not have a direct indicator of regional variation in need for these operations, we used the hospital district level musculoskeletal disorder index [27] as a proxy. The index presents the proportion of working age persons (16–64 years) who, at the end of the year, were on a disability pension due to a disorder of the musculoskeletal system (ICD10 codes M00-M99). The index is based on pension statistics of the Finnish Centre for Pensions and includes those receiving disability pension either under an earnings-related pension system, the national pension system, or both. Its regional values are calculated annually in proportion to the population insured by the Social Insurance Institution. As alternative area level factors examined in preliminary analyses, we calculated proportions of 65 + year-olds in each hospital district using the FOLK database as an indicator of population structure suggesting potential differences in need for services. Average temporal distances of the population of each municipality to hospitals performing hip and knee arthroplasties in their area were calculated in the University of Oulu from the Road and street network data provided by Esri Finland and the Finnish Transport Agency (Digiroad database)[28]. We further defined hospital district level average queueing time for these operations using information from the HILMO data base and the number of orthopaedic surgeons per population at the area obtained from the Finnish Medical Association [29] as indicators of supply of services. An additional file presents the effect of each of the different area-level variables to the models (both the Null Model including only age and gender and Model4 including all individual level variables examined) [see Additional file 1].

Ethical approval for the study was received from the Research Ethics Committee of THL. Permissions to use register data from Care Register and FAR were sought from THL and permission to use the FOLK data from Statistics Finland. All linkages were performed by competent authorities using the personal identification codes unique to each individual in Finland. The data was pseudonymised before handing it over to the research team to be used through the remote access system of Statistics Finland.

### Statistical methods

We built on analysis strategy presented by Falster et al. [30] and analysed the data with two-level multilevel Poisson regression models with hospital district as a random factor as we aimed to assess the extent of regional variation and how much of it and changes in it over time could be explained by both individual and area level factors. We used Markov chain Monte Carlo (MCMC) estimation techniques with 10 000 iterations and defined medians as estimates for variance and 2.5^th^ and 97.5^th^ percentiles as estimates for 95% credible intervals. We analysed the incidence of primary hip and knee arthroplasty separately in two consecutive time periods: 2010 − 2013 and 2014 − 2017 as the annual numbers of the procedures were not sufficiently high for the analyses.

By first analysing the age and gender adjusted model (Model 0) we acquired the estimate of variance (σ^2^) for hospital district which represents the baseline estimate for regional variation in the analysis. Subsequent models were compared to Model 0. We measured how each additional factor affected hospital district variance using the proportional change in variance (PCV) as a measure [31]. We added the factors into the models in the following order: comorbidity (Model 1), level of education (Model 2) occupational social class (Model 3), income quintile (Model 4), and hospital district level musculoskeletal disorder index (Model 5) as shown in an Additional file [see Additional file 2]. Incidence rate ratios (IRR) were calculated for both explanatory variables and hospital district estimates of the incidence of hip and knee arthroplasties to measure how well they predicted the outcomes. Additional file 3 shows the separate effect of each of the variables compared to the Null models. Finally, we calculated Median Rate Ratios (MRRs) [32] for the models. The MRRs were used to describe the relative differences in likelihoods to undergo these operations in different regions, after adjusting the models with explanatory factors.

## Results

**Fig. 1:**
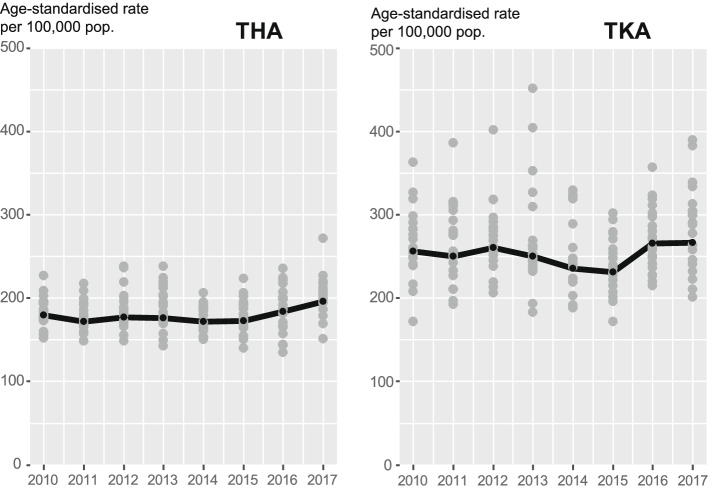
Annual rates (/100 000 person years) of elective total hip (THA) and knee (TKA) arthroplasties by hospital district in Finland between 2010 − 2017

Altogether 59 446 primary hip arthroplasties and 83 888 primary knee arthroplasties were performed in Finland in 2010 − 2017. For hip arthroplasties the numbers increased from 28 139 in 2010 − 2013 to 31 271 in 2014 − 2017 period. The numbers of knee arthroplasties increased from 40 620 to 43 242. In 2010 − 2013 the age-standardised rate of hip arthroplasties was 176/100 000 and in 2014 − 2017 181/100 000 person years. For knee arthroplasties the figures were 254 and 250/100 000 person years. Figure 1 presents annual age-adjusted rates per 100 000 person years in primary hip and knee arthroplasties throughout the study period. In the figure, the solid line represents whole country rates and each of the dots a hospital district. There was more hospital district variation in hip arthroplasties than for knee arthroplasties especially at the beginning of the study period. The whole country rates remained relatively stable throughout the study period.

Table 1 presents the basic background characteristics among patients with hip and knee arthroplasties. Both operations were more common among women, and the prevalence of operations increased with age up to 74 years. Approximately one fifth of those operated on had at least one comorbid condition. More than 40% of patients had basic education only. In both hip and knee replacements almost half of the patients belonged to the two lowest income quintiles in 2010 − 2013 and approximately 30% in 2014 − 2017. Substantial regional variation was found for the musculoskeletal disorder index suggesting regional differences in need.

**Table 1:** Basic background characteristics of the study population in hip (THA) and knee (TKA) arthroplasty in 2010 − 2013 and 2014 − 2017

		THA		TKA	
Variable	Category	2010 − 2013	2014 − 2017	2010 − 2013	2014 − 2017
Gender †	Women	56	57	66	65
Age (years)^a^	-49	5	5	3	3
	50–54	6	6	6	6
	55–59	10	10	11	11
	60–64	16	14	17	15
	65–69	18	20	17	20
	70–74	18	18	19	18
	75–79	15	15	16	15
	80 +	12	12	11	11
Comorbidities^a^	1	15	16	17	18
	2 +	4	5	4	5
Education^a^	Basic	46	38	49	39
	Secondary	31	34	33	38
	Higher	23	27	18	23
Occupation^a^	Higher white-collar	14	16	11	13
	Lower white-collar	30	32	31	34
	Blue-collar	36	34	39	36
	Farmer	10	8	10	7
	Other	10	10	9	10
Income	Lowest	18	15	18	14
	2	28	27	30	27
	3	21	21	21	23
	4	16	18	16	18
	Highest	17	19	15	18
Population need^b^	Musculoskeletal disorder index	78, 193	65, 166	78, 193	65, 166
Number of operations		28 139	31 271	40 620	43 242

^a^proportion

^b^range

**Fig. 2:**
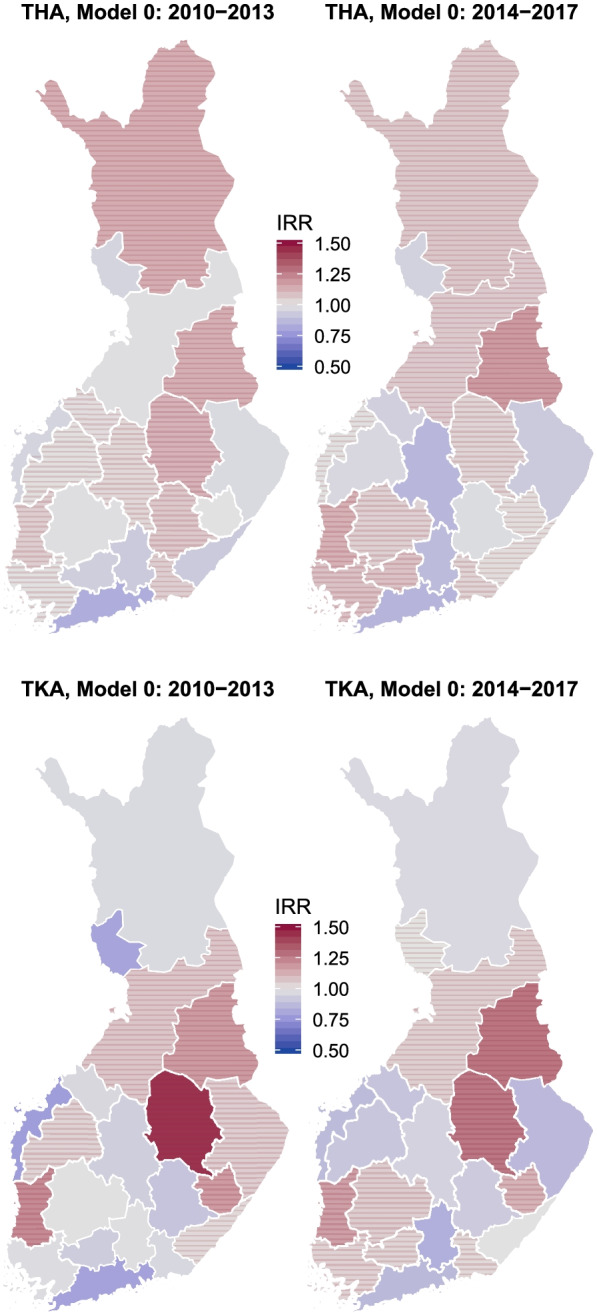
Hospital district variation of elective total hip (THA) and knee (TKA) arthroplasties controlling for age and gender only (Null model) in 2010 − 2013 and 2014 − 2017. Striped areas have incidence rate rations (IRR) over the country average

Figure 2 presents hospital district variation in age- and gender-adjusted IRRs for hip and knee arthroplasties. In this Figure, the incidence of arthroplasties was lower than the national average in the regions coloured blue (IRR < 1)– and higher in those coloured red (IRR > 1). Especially in hip operations IRRs were more often lower in southern and western Finland. There were larger variations in knee than in hip arthroplasties throughout the study period, but this difference diminished during the study period as the IRRs moved toward the national average in many of the hospital districts.

We first analysed hip arthroplasties in 2010 − 2013. In the final model (Model 5), all individual level factors, i.e., female gender, older age, having no or one comorbidity, basic or secondary education, occupation as a farmer and having higher than the lowest quintile income were associated with higher IRR in 2010 − 2013. Having two or more comorbidities, higher education or low income decreased the likelihood of the operation. We performed additional analysis adding each of the individual level factors to Model 0 separately, and the results were similar to those presented in Table 2 suggesting that the effect of each of these variables to IRR of operations was relatively independent. These results are presented in an Additional file [see Additional file 3]. Even after controlling for patient characteristics at individual level, high district level musculoskeletal disorder index indicating high population level need was associated with higher IRR for hip arthroplasties.

**Table 2:** Incidence rate ratios (IRR) and their 95% credible intervals (CI 95%) obtained from Markov chain Monte Carlo Poisson regression models for regional variation of hip (THA) and knee (TKA) arthroplasty in two time-periods

	THA 2010–2013	THA 2014–2017	TKA 2010–2013	TKA 2014–2017
Variable	IRR (CI 95%)	IRR (CI 95%)	IRR (CI 95%)	IRR (CI 95%)
Gender				
Male	1.00	1.00	1.00	1.00
Female	1.10 (1.08–1.13)	1.15 (1.13–1.18)	1.67 (1.63–1.71)	1.64 (1.61–1.68)
Age (years old)				
–49	1.00	1.00	1.00	1.00
50–54	4.76 (4.44–5.08)	5.68 (5.30–6.06)	7.15 (6.70–7.65)	7.40 (6.96–7.88)
55–59	8.53 (7.96–9.12)	9.40 (8.71–10.03)	13.74 (12.85–14.69)	14.16 (13.34–15.05)
60–64	12.35 (11.52–13.16)	12.66 (11.85–13.50)	18.68 (17.51–19.88)	17.80 (16.81–18.98)
65–69	17.70 (16.67–18.83)	18.25 (17.09–19.41)	23.67 (22.25–25.25)	22.94 (21.74–24.48)
70–74	23.80 (22.44–25.42)	22.79 (21.38–24.10)	33.74 (31.74–35.85)	28.44 (26.82–30.25)
75–79	25.72 (24.16–27.48)	25.65 (24.01–27.15)	35.41 (33.12–37.69)	31.05 (29.25–33.07)
80 +	14.65 (13.51–15.67)	15.93 (14.86–17.05)	18.49 (17.23–19.63)	16.76 (15.71–17.98)
No. comorbidities				
0	1.00	1.00	1.00	1.00
1	1.09 (1.05–1.14)	1.13 (1.10–1.17)	1.30 (1.26–1.34)	1.37 (1.32–1.40)
2 +	0.83 (0.78–0.88)	0.89 (0.83–0.95)	1.01 (0.95–1.06)	1.09 (1.03–1.14)
Education				
Lower	1.00	1.00	1.00	1.00
Secondary	0.99 (0.96–1.02)	0.99 (0.96–1.02)	0.97 (0.95–1.00)	1.03 (1.01–1.06)
Higher	0.93 (0.89–0.97)	0.94 (0.91–0.97)	0.70 (0.68–0.73)	0.80 (0.77–0.82)
Occupation				
Higher non-manual	1.00	1.00	1.00	1.00
Lower non-manual	0.99 (0.94–1.03)	1.03 (1.00–1.07)	1.07 (1.02–1.11)	1.11 (1.07–1.15)
Manual	1.00 (0.95–1.05)	0.99 (0.95–1.04)	1.13 (1.09–1.19)	1.15 (1.11–1.20)
Farmer	1.41 (1.34–1.51)	1.41 (1.35–1.49)	1.44 (1.37–1.53)	1.46 (1.38–1.54)
Other	0.99 (0.95–1.05)	1.00 (0.96–1.06)	1.03 (0.98–1.09)	1.05 (1.01–1.11)
Income quintile				
Highest	1.00	1.00	1.00	1.00
4	0.98 (0.94–1.02)	0.96 (0.92–1.00)	1.05 (1.01–1.09)	0.97 (0.93–1.00)
3	1.00 (0.96–1.04)	0.95 (0.92–0.99)	1.02 (0.98–1.06)	0.96 (0.93–1.00)
2	0.98 (0.94–1.02)	0.93 (0.90–0.97)	0.98 (0.94–1.01)	0.89 (0.86–0.92)
Lowest	0.81 (0.77–0.85)	0.72 (0.69–0.76)	0.74 (0.72–0.78)	0.64 (0.62–0.67)
Musculoskeletal disorder index (+ 1SD)	1.05 (1.02–1.09)	1.03 (0.98–1.08)	1.08 (1.01–1.15)	1.07 (1.01–1.13)

We then examined the effect of these variables in explaining regional differences in hip arthroplasties (Table 3). In gender and age-adjusted models, comorbidities explained 4% of regional variation, while individual socioeconomic variables (education, occupational social class and income) explained altogether an additional 9% of the variance between hospital districts. However, unlike other socioeconomic factors, adding income to the model in fact increased the variance, i.e. decreased the explanatory power of socioeconomic factors by 5%. This suggests that while on average the lowest income group had poorer access to operations when compared to higher income groups (as shown in Table 2), this effect varied between hospital districts. Indeed, in some hospital districts with the lowest IRRs the income distribution of arthroplasties favoured higher quintiles even more than on average – and in some districts with the highest IRRs this distribution was more favourable for those within the lowest quintile. Differences in the area level musculoskeletal disorder index explained a large part (44%) of the variance.

**Table 3:** Hospital district variance (σ^2^) and its 95% credible interval (CI 95%) in elective primary total hip and knee arthroplasty in Finland for years 2010 − 2013 and 2014 − 2017, the proportion of variance explained (PCV) and Median Rate Ratio (MRR) when adding variables to the models

	2010–2013			2014–2017		
Hip arthroplasty	σ2 (CI 95%)	PCV	MRR (CI 95%)	σ2 (CI 95%)	PCV	MRR (CI 95%)
M0 Null model	0.007 (0.003–0.015)	–	1.08 (1.06–1.12)	0.009 (0.004–0.019)	–	1.09 (1.06–1.14)
M1 + Comorbidity	0.007 (0.003–0.014)	4.0	1.08 (1.06–1.12)	0.009 (0.004–0.018)	0.3	1.09 (1.07–1.14)
M2 + Education	0.006 (0.003–0.013)	13.6	1.08 (1.05–1.12)	0.009 (0.004–0.019)	-0.2	1.09 (1.07–1.14)
M3 + Occupation	0.006 (0.003–0.012)	17.9	1.07 (1.05–1.11)	0.008 (0.004–0.018)	2.6	1.09 (1.06–1.14)
M4 + Income	0.006 (0.003–0.013)	13.3	1.08 (1.05–1.12)	0.009 (0.004–0.019)	-1.6	1.09 (1.07–1.14)
M5 + MSD Index	0.003 (0.001–0.007)	57.7	1.05 (1.03–1.08)	0.008 (0.004–0.018)	3.1	1.09 (1.06–1.14)
Knee arthroplasty						
M0 Null model	0.024 (0.013–0.051)	–	1.16 (1.11–1.24)	0.016 (0.008–0.033)	–	1.13 (1.09–1.19)
M1 + Comorbidity	0.024 (0.013–0.050)	-0.7	1.16 (1.11–1.24)	0.016 (0.009–0.034)	-4.9	1.13 (1.09–1.19)
M2 + Education	0.023 (0.012–0.048)	4.5	1.16 (1.11–1.23)	0.016 (0.009–0.035)	-4.7	1.13 (1.09–1.19)
M3 + Occupation	0.022 (0.012–0.046)	9.3	1.15 (1.11–1.23)	0.016 (0.009–0.033)	-2.2	1.13 (1.09–1.19)
M4 + Income	0.023 (0.013–0.049)	3.3	1.16 (1.11–1.24)	0.017 (0.009–0.036)	-9.0	1.13 (1.09–1.20)
M5 + MSD Index	0.018 (0.009–0.039)	25.4	1.14 (1.10–1.21)	0.013 (0.007–0.029)	15.7	1.12 (1.08–1.17)

In 2014 − 2017, individual level factors associated with hip arthroplasties were similar to those in 2010 − 2013. However, only higher level of education was statistically significantly associated to IRR of hip arthroplasties in the final model (Model 5). Farmers were more likely and those with lower income less likely to have undergone the operation. The lower the income the lower the likelihood of hip arthroplasty was. High area level musculoskeletal index did not have a statistically significant IRR that deviated from 1.00. When examining factors explaining regional variation (Table 3), individual socioeconomic position and comorbidity as well as area level musculoskeletal disorder index had little or no effect on regional variation, while the income effect was similar to that of the 2010 − 2013 period. An additional file presents maps showing regional variation in hip arthroplasties in both periods after controlling for all these factors [see Additional file 4].

In 2010 − 2013, the final model (Model 5) for knee arthroplasties suggested that all individual level factors were associated with IRR of the operation (Table 2). Female gender, older age, having zero or one comorbidity and either lower non-manual, blue-collar or farmer as occupational social class were associated with increased IRR of knee arthroplasty. Low income was associated with lower IRR of the operation. When comparing the effect of each of the socioeconomic variables, adding income to the model increased the IRRs for occupational social class somewhat also in these operations. Having two or more comorbidities did not have a significant IRR for knee arthroplasty and having higher education was associated with lower IRR of the operation. Adding socioeconomic variables to the model decreased the IRR of the operation especially in the oldest age groups (70 + years of age). At the area level, increased musculoskeletal disorder index was associated with higher IRR of the operation. When examining factors explaining regional differences (Table 3), individual level socioeconomic variables, i.e., education, occupational social class and income had limited effect (3%), while regional differences in need (musculoskeletal disorder index 22%) emerged as the most important explanatory factor. Similarly to hip arthroplasties in 2010–13, adding income to the model increased the variance by 6%.

In 2014 − 2017, individual level factors were mostly associated with IRR of knee arthroplasty in the same pattern as in the earlier time period (Table 2). Also in this period, adding socioeconomic factors to the models decreased the IRR of operation somewhat in the oldest age groups (75 +). In contrast to the results for 2010 − 2013, having two or more comorbidities was associated with increased IRR of the operation. The area-level musculoskeletal disorder index was also significantly associated with increased IRR of the operation. When examining factors explaining regional differences in knee arthroplasties (Table 3), individual comorbidities increased the variance by 5% and socioeconomic variables by an additional 4%. Regional differences in need (musculoskeletal disorder index 25%) emerged as the only factor explaining the variance. An additional file presents the regional variation of the operation in both periods after controlling for all these factors [see Additional file 5].

Overall, the regional variances were relatively small in both hip and knee arthroplasty on both time periods. While individual level factors explained a negligible proportion of these variances, the area level musculoskeletal disorder index was a somewhat stronger explanatory factor. However, the effect of the index diminished over time. Moreover, when measured with median rate ratios (MRR), we found relatively small regional heterogeneity in operation risks. This heterogeneity remained rather constant even after controlling for individual level sociodemographic factors and area level need.

## Discussion

We found small and over time relatively stable regional variation in elective primary total hip arthroplasty in Finland 2010 − 2017, while in primary total knee arthroplasty the variation was larger and decreased slightly during the study period. In 2010 − 2013 individual socioeconomic variables explained 10% of variation in hip and 4% in knee arthroplasty, an effect that did not emerge in 2014 − 2017. The area-level musculoskeletal disorder index reflecting the need for care explained in total a further 44% of the variation in hip operations in 2010 − 2013, but only 5% in 2014 − 2017 – and respectively 22% and 25% in knee operations. Our results are in line with earlier studies from different countries reporting regional variation in these procedures [3–10].

While our model for hip arthroplasty in 2010 − 2013 explained regional variation surprisingly well, PCVs in the operation in 2014 − 2017 remained rather low. This may result from changes in hospital district level procedure rates between the time periods as our set of explanatory variables was unable to capture a large part of variation in the latter period. In our study, comorbidity did not explain much regional variation in either hip or knee arthroplasty suggesting similar criteria concerning eligibility to be used in hospital districts across the country. However, socioeconomic position of the patients did have a role in explaining some regional differences in hip and in knee arthroplasties in the first period. These results are in line with earlier research from other countries [5, 8, 13] although one study from Denmark found no association between regional variation of arthroplasties and socioeconomic position [9]. Additionally, some studies have found that rates were higher among those with higher socioeconomic position [8, 13]. In our study both operations were more common among farmers and knee arthroplasty also among lower white-collar and blue-collar workers. These results are in line with earlier studies indicating that heavy work load would predispose for osteoarthrosis of the hip or knee [17–20]. We found that the effect of socioeconomic factors on regional variation decreased during the study period. One factor that could have at least some effect on this development is the centralisation of specialised health care that has been under way in Finland limiting the number of hospitals performing these operations during the latter part of the study period [33]. As fewer hospitals perform these operations the criteria used are likely to become more uniform.

Regional differences in need seem to play a further important part in regional differences as a high musculoskeletal disorder index in the district was an important explanatory factor for regional differences in both procedures throughout the study period. While the proportion of 65 + year-olds did seem to explain some of the variation, distance to the hospital, queueing times, having a university level hospital in the area, and/or numbers of orthopaedic surgeons did not seem to be as important. We used the musculoskeletal disorder index as a regional indicator of need as it fitted the models best. Similar results have also been reported in earlier studies concerning differences in need and supply of services [3, 4, 8]. For instance, Judge et al. [8] reported that distance did not play a role in regional variation of either total hip or knee arthroplasty procedure rates and that other supply side characteristics including hospital level had only modest effects in the UK.

A strength of our study was that we were able to use nationwide, representative register data covering all Finnish residents in 2010 − 2017. The data were collected from the nationwide Care Register for Health Care based on clinical diagnoses. The overall accuracy and coverage of the register has been reported to be good [34]. However, we observed a regional abnormality in the procedure rates: within a single hospital district over time these rates not only decreased, but their decrease also accelerated towards the end of the study period. After detailed inquiries, we found that this discrepancy was caused by local incompatibilities between procedure records and the Care Register. Therefore, we used the Finnish Arthroplasty Register to complement the Care Register data which seemed to correct the abnormality. Starting from 2014, this complementation significantly increased the number of procedures in this single hospital district but had almost no effects elsewhere: within this hospital district the total number of hip arthroplasties in 2010–2017 increased by 17% – and knee arthroplasties by 30%. As the FAR register data are collected from hospitals separate from those of the Care Register, we assume that the number of missing/misclassified cases in our data set is relatively small after this correction. We used the annual FOLK data set maintained by Statistics Finland to obtain individual level data for gender, age, municipality of residence, level of education, income and occupational social class for each individual. These data are based on population censuses and taxation data.

We acknowledge that the change in PCVs in hip arthroplasties over time limits the interpretation of the results: our hip arthroplasty models explained almost no variance in 2014 − 2017. This finding was similar when reanalysed with two-year periods. Our interpretation is that it occurred because of the low variances in the null models which meant that even minor changes in hip arthroplasty procedure rates could affect the explanatory power of models. Indeed, there were some changes in the distribution and order of hospital districts rates between 2010 − 2013 and 2014 − 2017. Moreover, the low variances were accompanied by relatively large uncertainty: the credible intervals for the variances overlapped between the time periods as well as between subsequent models.

While we did not have direct information concerning individual need for these operations, we used the musculoskeletal disorder index as an indirect proxy for area level need allowing us to take regional differences in need for operations into account. Nevertheless, as current care guidelines rely on perceived functional limitations in addition to clinical severity of osteoarthritis, direct measurement of need is not possible in a total population approach. Further, we did not have information of obesity or exercise habits among the population as the registers used in this study do not record information on these, and thus we cannot evaluate their role in the decisions to operate. The index is based on numbers of working age population receiving disability pension due to musculoskeletal diseases in each of the study years and while it does not focus on disability pensions due to osteoarthritis of hip or knee only, it seems to represent regional differences in these diseases better than individual level indicators used in this study. The role of the referring primary care providers is a complex issue. As we did not have information of the health care sector of the referring primary care provider (municipal health centre, occupational health care or private sector) it is not possible to assess the effect of private or occupational health care on access to these operations. On the one hand, all employed people in Finland have access to occupational health care, but its content tends to vary between industries and companies. A broader coverage for curative services tends to be available for employees in larger companies and white-collar workplaces. Moreover, supply of occupational health care is likely to be better in hospital districts containing more urban areas. On the other hand, those still in the workforce and thus having access to occupational health care tend to be younger and in better health. Use of private health care is likely to be associated with income level of the patients as its use is only partially reimbursed by the national health insurance system. There is also likely to be regional variation in supply of private services. [22]

Our results suggest that eligibility criteria in total hip and total knee arthroplasty are increasingly consistent between Finnish hospital districts – only some regional differences emerged in both operations across the country in 2010 − 2017. At the individual level, factors related to need (older age and occupation including heavy physical workload) as well as socioeconomic factors (education and income) were associated with the likelihood of both operations. Our results further suggest that poorer access to both operations among those with lower income varies between hospital districts in both time periods. Regional differences in socioeconomic inequities in access to hip and knee arthroplasty would be an interesting question for further studies. However, the effect of individual socioeconomic position on regional differences was limited and decreased during the study period. Though two or more chronic comorbidities decreased the likelihood of total hip arthroplasty, this factor did not much affect regional variation in either operation. Contextual need related factors, measured in our study by regional musculoskeletal disease prevalence, seem to play an important role in regional differences in hip and knee arthroplasty. As the current regional equity in access to hip and knee arthroplasty appears promising, the next step should be to analyse regional equity in both quality and health outcomes of these operations. Another approach is to address the impact of individual level socioeconomic and health related factors which seems to associate with poor access to endoprosthesis surgery. This would be especially important in Finland where potential effects of policy actions including centralisation of specialised health care on regional and socioeconomic differences remain understudied.

## Conclusions

Our results suggest that eligibility criteria in total hip and knee arthroplasty are increasingly consistent between Finnish hospital districts. Factors related to individual level and regional level need both had an important role in explaining regional variations. Further study is needed on the effect of health policy on equity in access to care in these operations.

## Supplementary Information

**Additional file 1.** Hospital district variance (σ^2^) in elective primary total hip and knee arthroplasty in Finland for years 2010−2013 and 2014−2017, the proportion of variance explained (PCV) and Median Rate Ratio (MRR) when adding area-level variables one by one to both null model (M0) and the model with all individual variables (M4).

**Additional file 2.** Individual and area-level factors added into the Models – the subsequent Models include also factors from previous ones.

**Additional file 3.** Building of the two-level Poisson models for hip (THA) and knee (TKA) arthroplasty in two time periods.

**Additional file 4.** Hospital district variation of elective total hip (THA) arthroplasties controlling for age and gender only (Models 4 and 5) in 2010-2013 and 2014-2017. Striped areas have incidence rate rations (IRR) over the country average.

**Additional file 5.** Hospital district variation of elective total knee (TKA) arthroplasties controlling for age and gender only (Models 4 and 5) in 2010-2013 and 2014-2017. Striped areas have incidence rate rations (IRR) over the country average.

## Data Availability

The data that support the findings of this study are available from the Finnish Institute for Health and Welfare and Statistics Finland, but restrictions apply to the availability of the data used under license for the current study, and so they are not publicly available due to Finnish data protection legislation. According to the legislation, register authorities give permissions to use register data including sensitive individual information (e.g., health data) to study specified research questions to named individuals who have signed a pledge of secrecy and they are not permitted to forward it to other researchers. Other researchers can apply for the data from the Health and Social Data Permit Authority Findata. Findata handles the data permit applications concerning Finnish Institute for Health and Welfare registers (the Care Register for Health Care for operations and diagnoses in the current study), and Statistics Finland data when combined to other registers (population at risk and demographics) see https://www.findata.fi/en/services/data-requests/.

## Abbreviations

THA: Total elective hip arthroplasty
TKA: Total elective knee arthroplasty
IRR: Incidence rate ratio
PCV: Proportion of variance explained
MRR: Median rate ratio
MCMC: Markov chain Monte Carlo method
FAR: Finnish Arthroplasty Register
