# Supplementary material for: Explaining regional variation in elective hip and knee arthroplasties in Finland 2010 − 2017—a register-based cohort study
Source: BMC Health Serv Res. 2022 Jul 9;22:891. doi: 10.1186/s12913-022-08305-7 (PMC9270793; doi:10.1186/s12913-022-08305-7)
Supplement: Supplementary file 1 — Additional file 1. Hospital district variance (σ2) in elective primary total hip and knee arthroplasty in Finland for years 2010−2013 and 2014−2017, the proportion of variance explained (PCV) and Median Rate Ratio (MRR) when adding area-level variables one by one to both null model (M0) and the model with all individual variables (M4). [file 12913_2022_8305_MOESM1_ESM.docx]

Additional file 1**.** Hospital district variance (σ^2^) in elective primary total hip and knee arthroplasty in Finland for years 2010−2013 and 2014−2017, the proportion of variance explained (PCV) and Median Rate Ratio (MRR) when adding area-level variables one by one to both null model (M0) and the model with all individual variables (M4).

|  | 2010–2013 | | |  | 2014–2017 | | |
| --- | --- | --- | --- | --- | --- | --- | --- |
| Hip replacement | σ^2^ (CI 95 %) | PCV | MRR (CI 95 %) |  | σ^2^ (CI 95 %) | PCV | MRR (CI 95 %) |
| M0 Null model | 0.007 (0.003–0.015) | – | 1.08 (1.06–1.12) |  | 0.009 (0.004–0.019) | – | 1.09 (1.06–1.14) |
| M0 + MSD Index | 0.003 (0.001–0.008) | 56.0 | 1.05 (1.03–1.09) |  | 0.008 (0.004–0.019) | 3.5 | 1.09 (1.06–1.14) |
| M0 + Proportion of Population Aged +65 | 0.005 (0.002–0.012) | 25.1 | 1.07 (1.05–1.11) |  | 0.008 (0.004–0.018) | 6.9 | 1.09 (1.06–1.14) |
| M0 + Mean Distance to Hospital (km) | 0.005 (0.003–0.012) | 19.5 | 1.07 (1.05–1.11) |  | 0.009 (0.004–0.019) | -1.9 | 1.09 (1.07–1.14) |
| M0 + Number of Orthopaedics | 0.006 (0.003–0.014) | 12.1 | 1.08 (1.05–1.12) |  | 0.009 (0.004–0.019) | -2.0 | 1.09 (1.07–1.14) |
| M0 + Queueing Time | 0.006 (0.003–0.015) | 5.9 | 1.08 (1.05–1.12) |  | 0.009 (0.004–0.020) | -2.3 | 1.09 (1.07–1.14) |
| M0 + University Hospital | 0.007 (0.003–0.015) | 1.9 | 1.08 (1.06–1.12) |  | 0.009 (0.005–0.020) | -4.2 | 1.09 (1.07–1.14) |
|  |  |  |  |  |  |  |  |
| M0 Null model | 0.007 (0.003–0.015) | – | 1.08 (1.06–1.12) |  | 0.009 (0.004–0.019) | – | 1.09 (1.06–1.14) |
| M4: M0 + Comorbidities + Individual SEP | 0.006 (0.003–0.013) | 13.3 | 1.08 (1.05–1.12) |  | 0.009 (0.004–0.019) | -1.6 | 1.09 (1.07–1.14) |
| M4 + MSD Index | 0.003 (0.001–0.007) | 60.2 | 1.05 (1.03–1.08) |  | 0.008 (0.004–0.018) | 6,2 | 1.09 (1.06–1.14) |
| M4 + Proportion of Population Aged +65 | 0.005 (0.003–0.012) | 22.0 | 1.07 (1.05–1.11) |  | 0.009 (0.005–0.020) | -3,4 | 1.09 (1.07–1.14) |
| M4 + Mean Distance to Hospital (km) | 0.005 (0.002–0.011) | 27.9 | 1.07 (1.05–1.11) |  | 0.009 (0.005–0.021) | -6.9 | 1.10 (1.07–1.15) |
| M4 + Number of Orthopaedics | 0.006 (0.003–0.013) | 19.1 | 1.07 (1.05–1.11) |  | 0.009 (0.005–0.021) | -7,9 | 1.10 (1.07–1.15) |
| M4 + Queueing Time | 0.006 (0.003–0.013) | 14.7 | 1.08 (1.05–1.11) |  | 0.009 (0.005–0.020) | -3,1 | 1.09 (1.07–1.14) |
| M4 + University Hospital | 0.006 (0.003–0.015) | 6.2 | 1.08 (1.05–1.12) |  | 0.009 (0.005–0.020) | -9,2 | 1.10 (1.07–1.15) |

|  | 2010–2013 | | |  | 2014–2017 | | |
| --- | --- | --- | --- | --- | --- | --- | --- |
| Knee replacement | σ^2^ (CI 95 %) | PCV | MRR (CI 95 %) |  | σ^2^ (CI 95 %) | PCV | MRR (CI 95 %) |
| M0 Null model | 0.024 (0.013–0.051) | – | 1.16 (1.11–1.24) |  | 0.016 (0.008–0.033) | – | 1.13 (1.09–1.19) |
| M0 + MSD Index | 0.017 (0.009–0.038) | 28.8 | 1.13 (1.09–1.20) |  | 0.012 (0.006–0.026) | 23.2 | 1.11 (1.08–1.17) |
| M0 + Proportion of Population Aged +65 | 0.022 (0.012–0.047) | 9.5 | 1.15 (1.11–1.23) |  | 0.015 (0.008–0.033) | 2.9 | 1.13 (1.09–1.19) |
| M0 + Mean Distance to Hospital (km) | 0.025 (0.013–0.054) | -5.1 | 1.16 (1.12–1.25) |  | 0.016 (0.009–0.034) | -3.0 | 1.13 (1.09–1.19) |
| M0 + Number of Orthopaedics | 0.025 (0.014–0.054) | -5.8 | 1.16 (1.12–1.25) |  | 0.016 (0.009–0.036) | -4.3 | 1.13 (1.09–1.20) |
| M0 + Queueing Time | 0.025 (0.013–0.053) | -3.7 | 1.16 (1.12–1.25) |  | 0.016 (0.008–0.034) | -1.0 | 1.13 (1.09–1.19) |
| M0 + University Hospital | 0.025 (0.013–0.053) | -3.6 | 1.16 (1.11–1.24) |  | 0.015 (0.008–0.033) | 2.9 | 1.13 (1.09–1.19) |
|  |  |  |  |  |  |  |  |
| M0 Null model | 0.024 (0.013–0.051) | – | 1.16 (1.11–1.24) |  | 0.016 (0.008–0.033) | – | 1.13 (1.09–1.19) |
| M4: M0 + Comorbidities + Individual SEP | 0.023 (0.013–0.049) | 3.3 | 1.16 (1.11–1.24) |  | 0.017 (0.009–0.036) | -9.0 | 1.13 (1.09–1.20) |
| M4 + MSD Index | 0.017 (0.009–0.037) | 30.1 | 1.13 (1.09–1.20) |  | 0.013 (0.007–0.028) | 16.9 | 1.12 (1.08–1.17) |
| M4 + Proportion of Population Aged +65 | 0.021 (0.011–0.045) | 13.0 | 1.15 (1.11–1.22) |  | 0.016 (0.009–0.036) | -5.0 | 1.13 (1.09–1.20) |
| M4 + Mean Distance to Hospital (km) | 0.024 (0.013–0.052) | -1.2 | 1.16 (1.11–1.24) |  | 0.018 (0.009–0.038) | -14.0 | 1.14 (1.10–1.20) |
| M4 + Number of Orthopaedics | 0.024 (0.013–0.053) | -0.2 | 1.16 (1.11–1.25) |  | 0.018 (0.009–0.039) | -13.3 | 1.14 (1.10–1.21) |
| M4 + Queueing Time | 0.024 (0.013–0.051) | -1.0 | 1.16 (1.11–1.24) |  | 0.017 (0.009–0.037) | -9.5 | 1.13 (1.10–1.20) |
| M4 + University Hospital | 0.024 (0.013–0.053) | -0.6 | 1.16 (1.11–1.24) |  | 0.017 (0.009–0.037) | -10.2 | 1.13 (1.10–1.20) |
