# Supplementary material for: Explaining regional variation in elective hip and knee arthroplasties in Finland 2010 − 2017—a register-based cohort study
Source: BMC Health Serv Res. 2022 Jul 9;22:891. doi: 10.1186/s12913-022-08305-7 (PMC9270793; doi:10.1186/s12913-022-08305-7)
Supplement: Supplementary file 2 — Additional file 2. Individual and area-level factors added into the Models – the subsequent Models include also factors from previous ones. [file 12913_2022_8305_MOESM2_ESM.docx]

Additional file 2. Individual and area-level factors added into the Models – the subsequent Models include also factors from previous ones

| Added to | Factors | Hypothesized pathway for risk of arthroplasty |
| --- | --- | --- |
| **Individual level** | | |
| Model 0 | Age and gender | Null model with individual demographics |
| Model 1 | Saver’s index of comorbidities | Multimorbidity may complicate treatments or act as a relative contraindication |
| Model 2 | Level of education | Health literacy |
| Model 3 | Lifetime occupational social class | Physical workload predisposes to osteoarthrosis |
| Model 4 | Income quintile | Material resources |
| **Area-level** | | |
| Model 5 | Musculoskeletal disorder index | Differences in musculoskeletal diseases reflect need for orthopaedic care in general and may explain differences in use between hospital districts |
