# Supplementary material for: Explaining regional variation in elective hip and knee arthroplasties in Finland 2010 − 2017—a register-based cohort study
Source: BMC Health Serv Res. 2022 Jul 9;22:891. doi: 10.1186/s12913-022-08305-7 (PMC9270793; doi:10.1186/s12913-022-08305-7)
Supplement: Supplementary file 3 — Additional file 3. Building of the two-level Poisson models for hip (THA) and knee (TKA) arthroplasty in two time periods. [file 12913_2022_8305_MOESM3_ESM.docx]

Additional file 3. Incidence rate ratios (IRR) and their 95 % credible intervals (CI 95 %) obtained from Markov chain Monte Carlo Poisson regression models for regional variation of hip (THA) and knee (TKA) arthroplasty in two time-periods.

| THA 2010–2013 | Model 0 | Model 1 | Model 2 | Model 3 | Model 4 | Model 5 |
| --- | --- | --- | --- | --- | --- | --- |
| Variable | IRR (CI 95%) | IRR (CI 95%) | IRR (CI 95%) | IRR (CI 95%) | IRR (CI 95%) | IRR (CI 95%) |
| Gender |  |  |  |  |  |  |
| Male | 1.00 | 1.00 | 1.00 | 1.00 | 1.00 | 1.00 |
| Female | 1.08 (1.05–1.11) | 1.08 (1.05–1.11) | 1.08 (1.05–1.11) | 1.09 (1.06–1.12) | 1.10 (1.08–1.13) | 1.10 (1.08–1.13) |
| Age (years old) |  |  |  |  |  |  |
| 45–49 | 1.00 | 1.00 | 1.00 | 1.00 | 1.00 | 1.00 |
| 50–54 | 4.76 (4.41–5.12) | 4.84 (4.51–5.16) | 4.78 (4.40–5.26) | 4.76 (4.48–5.09) | 4.77 (4.40–5.13) | 4.76 (4.44–5.08) |
| 55–59 | 8.56 (8.04–9.11) | 8.64 (8.12–9.17) | 8.57 (7.97–9.17) | 8.55 (8.09–9.10) | 8.55 (7.87–9.30) | 8.53 (7.96–9.12) |
| 60–64 | 12.53 (11.82–13.25) | 12.60 (11.75–13.41) | 12.47 (11.75–13.25) | 12.50 (11.86–13.23) | 12.45 (11.63–13.37) | 12.35 (11.52–13.16) |
| 65–69 | 18.18 (17.14–19.22) | 18.21 (17.20–19.33) | 18.03 (16.89–19.14) | 17.99 (17.09–18.97) | 17.91 (16.72–19.22) | 17.70 (16.67–18.83) |
| 70–74 | 24.53 (23.14–25.83) | 24.57 (23.16–26.14) | 24.26 (22.65–25.81) | 23.96 (22.74–25.24) | 23.94 (22.26–25.95) | 23.80 (22.44–25.42) |
| 75–79 | 26.41 (25.05–27.98) | 26.61 (25.10–28.31) | 26.10 (24.50–27.81) | 25.63 (24.12–27.30) | 25.94 (24.32–28.16) | 25.72 (24.16–27.48) |
| 80+ | 14.77 (13.90–15.78) | 14.95 (13.91–15.98) | 14.62 (13.60–15.71) | 14.17 (13.39–14.99) | 14.80 (13.86–15.84) | 14.65 (13.51–15.67) |
| No. comorbidities |  |  |  |  |  |  |
| 0 |  | 1.00 | 1.00 | 1.00 | 1.00 | 1.00 |
| 1 |  | 1.08 (1.05–1.12) | 1.08 (1.05–1.11) | 1.08 (1.04–1.12) | 1.09 (1.05–1.14) | 1.09 (1.05–1.14) |
| 2+ |  | 0.82 (0.77–0.88) | 0.81 (0.76–0.85) | 0.81 (0.76–0.86) | 0.81 (0.76–0.86) | 0.83 (0.78–0.88) |
| Education |  |  |  |  |  |  |
| Lower |  |  | 1.00 | 1.00 | 1.00 | 1.00 |
| Secondary |  |  | 0.99 (0.96–1.03) | 1.00 (0.98–1.03) | 0.99 (0.96–1.03) | 0.99 (0.96–1.02) |
| Higher |  |  | 0.93 (0.91–0.96) | 0.95 (0.91–0.99) | 0.94 (0.90–0.98) | 0.93 (0.89–0.97) |
| Occupation |  |  |  |  |  |  |
| Higher non-manual |  |  |  | 1.00 | 1.00 | 1.00 |
| Lower non-manual |  |  |  | 0.99 (0.95–1.03) | 0.99 (0.96–1.03) | 0.99 (0.94–1.03) |
| Manual |  |  |  | 0.98 (0.93–1.03) | 1.00 (0.96–1.06) | 1.00 (0.95–1.05) |
| Farmer |  |  |  | 1.35 (1.27–1.42) | 1.42 (1.34–1.48) | 1.41 (1.34–1.51) |
| Other |  |  |  | 0.96 (0.92–1.01) | 1.01 (0.95–1.06) | 0.99 (0.95–1.05) |
| Income quintile |  |  |  |  |  |  |
| Highest |  |  |  |  | 1.00 | 1.00 |
| 4 |  |  |  |  | 0.98 (0.94–1.02) | 0.98 (0.94–1.02) |
| 3 |  |  |  |  | 1.00 (0.96–1.05) | 1.00 (0.96–1.04) |
| 2 |  |  |  |  | 0.98 (0.95–1.03) | 0.98 (0.94–1.02) |
| Lowest |  |  |  |  | 0.81 (0.78–0.86) | 0.81 (0.77–0.85) |
| Musculoskeletal disorder index (+1SD) |  |  |  |  |  | 1.05 (1.02–1.09) |

| THA 2014–2017 | Model 0 | Model 1 | Model 2 | Model 3 | Model 4 | Model 5 |
| --- | --- | --- | --- | --- | --- | --- |
| Variable | IRR (CI 95%) | IRR (CI 95%) | IRR (CI 95%) | IRR (CI 95%) | IRR (CI 95%) | IRR (CI 95%) |
| Gender |  |  |  |  |  |  |
| Male | 1.00 | 1.00 | 1.00 | 1.00 | 1.00 | 1.00 |
| Female | 1.13 (1.10–1.16) | 1.13 (1.10–1.16) | 1.13 (1.11–1.16) | 1.12 (1.10–1.15) | 1.15 (1.12–1.18) | 1.15 (1.13–1.18) |
| Age (years old) |  |  |  |  |  |  |
| 45–49 | 1.00 | 1.00 | 1.00 | 1.00 | 1.00 | 1.00 |
| 50–54 | 5.71 (5.36–6.27) | 5.76 (5.34–6.17) | 5.76 (5.36–6.22) | 5.74 (5.30–6.11) | 5.65 (5.28–6.12) | 5.68 (5.30–6.06) |
| 55–59 | 9.56 (8.97–10.41) | 9.60 (9.07–10.22) | 9.61 (8.99–10.37) | 9.57 (8.98–10.28) | 9.35 (8.69–10.02) | 9.40 (8.71–10.03) |
| 60–64 | 13.01 (12.28–14.00) | 13.01 (12.29–13.79) | 13.04 (12.26–14.01) | 12.93 (12.12–13.83) | 12.68 (11.84–13.58) | 12.66 (11.85–13.50) |
| 65–69 | 18.83 (17.69–20.28) | 18.71 (17.72–19.82) | 18.75 (17.76–20.08) | 18.52 (17.41–19.68) | 18.16 (17.11–19.43) | 18.25 (17.09–19.41) |
| 70–74 | 23.39 (22.07–25.30) | 23.30 (22.12–24.62) | 23.30 (22.01–25.01) | 22.94 (21.59–24.46) | 22.57 (21.33–24.19) | 22.79 (21.38–24.10) |
| 75–79 | 26.38 (24.68–28.31) | 26.12 (24.68–27.74) | 26.15 (24.62–28.07) | 25.47 (23.51–26.95) | 25.37 (23.77–27.05) | 25.65 (24.01–27.15) |
| 80+ | 16.12 (15.05–17.41) | 16.06 (15.07–16.97) | 15.98 (15.03–17.32) | 15.45 (14.37–16.59) | 15.91 (14.72–16.94) | 15.93 (14.86–17.05) |
| No. comorbidities |  |  |  |  |  |  |
| 0 |  | 1.00 | 1.00 | 1.00 | 1.00 | 1.00 |
| 1 |  | 1.12 (1.08–1.16) | 1.11 (1.08–1.15) | 1.12 (1.09–1.16) | 1.13 (1.09–1.17) | 1.13 (1.10–1.17) |
| 2+ |  | 0.88 (0.83–0.92) | 0.87 (0.82–0.91) | 0.88 (0.82–0.92) | 0.89 (0.85–0.93) | 0.89 (0.83–0.95) |
| Education |  |  |  |  |  |  |
| Lower |  |  | 1.00 | 1.00 | 1.00 | 1.00 |
| Secondary |  |  | 1.01 (0.98–1.04) | 1.01 (0.98–1.04) | 0.99 (0.96–1.02) | 0.99 (0.96–1.02) |
| Higher |  |  | 0.98 (0.95–1.01) | 0.98 (0.95–1.01) | 0.94 (0.90–0.97) | 0.94 (0.91–0.97) |
| Occupation |  |  |  |  |  |  |
| Higher non-manual |  |  |  | 1.00 | 1.00 | 1.00 |
| Lower non-manual |  |  |  | 1.01 (0.97–1.06) | 1.03 (0.98–1.07) | 1.03 (1.00–1.07) |
| Manual |  |  |  | 0.95 (0.91–0.99) | 0.99 (0.95–1.04) | 0.99 (0.95–1.04) |
| Farmer |  |  |  | 1.31 (1.23–1.39) | 1.40 (1.33–1.48) | 1.41 (1.35–1.49) |
| Other |  |  |  | 0.94 (0.90–0.99) | 1.00 (0.95–1.06) | 1.00 (0.96–1.06) |
| Income quintile |  |  |  |  |  |  |
| Highest |  |  |  |  | 1.00 | 1.00 |
| 4 |  |  |  |  | 0.96 (0.93–1.00) | 0.96 (0.92–1.00) |
| 3 |  |  |  |  | 0.96 (0.92–1.00) | 0.95 (0.92–0.99) |
| 2 |  |  |  |  | 0.94 (0.90–0.98) | 0.93 (0.90–0.97) |
| Lowest |  |  |  |  | 0.72 (0.69–0.76) | 0.72 (0.69–0.76) |
| Musculoskeletal disorder index (+1SD) |  |  |  |  |  | 1.03 (0.98–1.08) |

| TKA 2010–2013 | Model 0 | Model 1 | Model 2 | Model 3 | Model 4 | Model 5 |
| --- | --- | --- | --- | --- | --- | --- |
| Variable | IRR (CI 95%) | IRR (CI 95%) | IRR (CI 95%) | IRR (CI 95%) | IRR (CI 95%) | IRR (CI 95%) |
| Gender |  |  |  |  |  |  |
| Male | 1.00 | 1.00 | 1.00 | 1.00 | 1.00 | 1.00 |
| Female | 1.63 (1.59–1.67) | 1.63 (1.59–1.67) | 1.62 (1.58–1.66) | 1.63 (1.59–1.67) | 1.67 (1.63–1.71) | 1.67 (1.63–1.71) |
| Age (years old) |  |  |  |  |  |  |
| 45–49 | 1.00 | 1.00 | 1.00 | 1.00 | 1.00 | 1.00 |
| 50–54 | 7.32 (6.78–7.87) | 7.39 (6.82–7.94) | 7.28 (6.67–7.90) | 7.20 (6.74–7.66) | 7.20 (6.68–7.82) | 7.15 (6.70–7.65) |
| 55–59 | 14.39 (13.48–15.41) | 14.35 (13.40–15.39) | 14.03 (12.90–15.05) | 13.83 (12.98–14.73) | 13.79 (12.84–14.87) | 13.74 (12.85–14.69) |
| 60–64 | 20.12 (18.86–21.41) | 19.83 (18.57–21.28) | 19.15 (17.83–20.40) | 18.88 (17.87–19.99) | 18.78 (17.63–20.18) | 18.68 (17.51–19.88) |
| 65–69 | 25.85 (24.27–27.49) | 25.32 (23.64–27.07) | 24.35 (22.62–25.93) | 23.98 (22.61–25.30) | 23.85 (22.51–25.59) | 23.67 (22.25–25.25) |
| 70–74 | 37.21 (34.83–39.67) | 36.07 (33.80–38.66) | 34.38 (31.90–36.61) | 33.60 (31.76–35.54) | 33.84 (31.76–36.31) | 33.74 (31.74–35.85) |
| 75–79 | 39.33 (36.98–42.09) | 37.81 (35.25–40.48) | 35.70 (33.05–37.89) | 34.66 (32.43–36.73) | 35.61 (33.59–38.40) | 35.41 (33.12–37.69) |
| 80+ | 20.43 (18.97–21.77) | 19.31 (17.93–20.79) | 18.03 (16.60–19.33) | 17.44 (16.38–18.51) | 18.60 (17.46–19.93) | 18.49 (17.23–19.63) |
| No. comorbidities |  |  |  |  |  |  |
| 0 |  | 1.00 | 1.00 | 1.00 | 1.00 | 1.00 |
| 1 |  | 1.33 (1.29–1.37) | 1.29 (1.26–1.33) | 1.29 (1.25–1.33) | 1.30 (1.26–1.34) | 1.30 (1.26–1.34) |
| 2+ |  | 1.03 (0.98–1.10) | 0.98 (0.93–1.03) | 0.98 (0.93–1.04) | 1.00 (0.94–1.05) | 1.01 (0.95–1.06) |
| Education |  |  |  |  |  |  |
| Lower |  |  | 1.00 | 1.00 | 1.00 | 1.00 |
| Secondary |  |  | 0.98 (0.95–1.01) | 0.99 (0.97–1.02) | 0.97 (0.95–1.00) | 0.97 (0.95–1.00) |
| Higher |  |  | 0.69 (0.67–0.71) | 0.73 (0.70–0.76) | 0.70 (0.68–0.73) | 0.70 (0.68–0.73) |
| Occupation |  |  |  |  |  |  |
| Higher non-manual |  |  |  | 1.00 | 1.00 | 1.00 |
| Lower non-manual |  |  |  | 1.06 (1.02–1.11) | 1.07 (1.03–1.11) | 1.07 (1.02–1.11) |
| Manual |  |  |  | 1.10 (1.05–1.15) | 1.14 (1.09–1.19) | 1.13 (1.09–1.19) |
| Farmer |  |  |  | 1.34 (1.28–1.42) | 1.45 (1.38–1.52) | 1.44 (1.37–1.53) |
| Other |  |  |  | 0.97 (0.93–1.02) | 1.04 (0.98–1.09) | 1.03 (0.98–1.09) |
| Income quintile |  |  |  |  |  |  |
| Highest |  |  |  |  | 1.00 | 1.00 |
| 4 |  |  |  |  | 1.05 (1.01–1.09) | 1.05 (1.01–1.09) |
| 3 |  |  |  |  | 1.02 (0.98–1.06) | 1.02 (0.98–1.06) |
| 2 |  |  |  |  | 0.98 (0.94–1.01) | 0.98 (0.94–1.01) |
| Lowest |  |  |  |  | 0.74 (0.71–0.78) | 0.74 (0.72–0.78) |
| Musculoskeletal disorder index (+1SD) |  |  |  |  |  | 1.08 (1.01–1.15) |

| TKA 2014–2017 | Model 0 | Model 1 | Model 2 | Model 3 | Model 4 | Model 5 |
| --- | --- | --- | --- | --- | --- | --- |
| Variable | IRR (CI 95%) | IRR (CI 95%) | IRR (CI 95%) | IRR (CI 95%) | IRR (CI 95%) | IRR (CI 95%) |
| Gender |  |  |  |  |  |  |
| Male | 1.00 | 1.00 | 1.00 | 1.00 | 1.00 | 1.00 |
| Female | 1.59 (1.56–1.63) | 1.59 (1.55–1.63) | 1.60 (1.56–1.63) | 1.59 (1.56–1.63) | 1.64 (1.60–1.68) | 1.64 (1.61–1.68) |
| Age (years old) |  |  |  |  |  |  |
| 45–49 | 1.00 | 1.00 | 1.00 | 1.00 | 1.00 | 1.00 |
| 50–54 | 7.55 (7.02–8.23) | 7.61 (7.09–8.20) | 7.65 (7.08–8.15) | 7.54 (7.04–8.11) | 7.32 (6.88–7.84) | 7.40 (6.96–7.88) |
| 55–59 | 14.88 (13.89–16.03) | 14.81 (13.94–16.02) | 14.74 (13.77–15.75) | 14.53 (13.68–15.59) | 14.04 (13.12–14.80) | 14.16 (13.34–15.05) |
| 60–64 | 19.07 (17.89–20.45) | 18.80 (17.68–20.03) | 18.58 (17.38–19.91) | 18.30 (17.10–19.53) | 17.69 (16.61–18.64) | 17.80 (16.81–18.98) |
| 65–69 | 24.69 (23.17–26.51) | 24.02 (22.65–25.72) | 23.72 (22.38–25.34) | 23.29 (21.93–24.89) | 22.72 (21.33–24.00) | 22.94 (21.74–24.48) |
| 70–74 | 30.41 (28.68–32.72) | 29.42 (27.68–31.45) | 29.11 (27.38–31.08) | 28.52 (26.97–30.39) | 28.03 (26.43–29.71) | 28.44 (26.82–30.25) |
| 75–79 | 33.42 (31.31–35.85) | 31.89 (30.03–34.04) | 31.41 (29.40–33.52) | 30.57 (28.55–32.51) | 30.60 (28.84–32.36) | 31.05 (29.25–33.07) |
| 80+ | 18.03 (16.89–19.37) | 16.83 (15.75–17.89) | 16.48 (15.38–17.63) | 15.95 (14.83–17.04) | 16.58 (15.59–17.61) | 16.76 (15.71–17.98) |
| No. comorbidities |  |  |  |  |  |  |
| 0 |  | 1.00 | 1.00 | 1.00 | 1.00 | 1.00 |
| 1 |  | 1.37 (1.33–1.41) | 1.36 (1.32–1.39) | 1.35 (1.31–1.39) | 1.36 (1.33–1.41) | 1.37 (1.32–1.40) |
| 2+ |  | 1.10 (1.05–1.15) | 1.07 (1.02–1.12) | 1.07 (1.02–1.12) | 1.09 (1.04–1.14) | 1.09 (1.03–1.14) |
| Education |  |  |  |  |  |  |
| Lower |  |  | 1.00 | 1.00 | 1.00 | 1.00 |
| Secondary |  |  | 1.05 (1.02–1.08) | 1.06 (1.03–1.08) | 1.03 (1.01–1.06) | 1.03 (1.01–1.06) |
| Higher |  |  | 0.82 (0.79–0.84) | 0.85 (0.82–0.88) | 0.80 (0.77–0.82) | 0.80 (0.77–0.82) |
| Occupation |  |  |  |  |  |  |
| Higher non-manual |  |  |  | 1.00 | 1.00 | 1.00 |
| Lower non-manual |  |  |  | 1.09 (1.05–1.14) | 1.11 (1.07–1.16) | 1.11 (1.07–1.15) |
| Manual |  |  |  | 1.09 (1.04–1.13) | 1.15 (1.10–1.20) | 1.15 (1.11–1.20) |
| Farmer |  |  |  | 1.32 (1.26–1.40) | 1.46 (1.39–1.54) | 1.46 (1.38–1.54) |
| Other |  |  |  | 0.97 (0.93–1.02) | 1.05 (1.00–1.10) | 1.05 (1.01–1.11) |
| Income quintile |  |  |  |  |  |  |
| Highest |  |  |  |  | 1.00 | 1.00 |
| 4 |  |  |  |  | 0.97 (0.94–1.01) | 0.97 (0.93–1.00) |
| 3 |  |  |  |  | 0.97 (0.93–1.00) | 0.96 (0.93–1.00) |
| 2 |  |  |  |  | 0.89 (0.86–0.93) | 0.89 (0.86–0.92) |
| Lowest |  |  |  |  | 0.65 (0.62–0.67) | 0.64 (0.62–0.67) |
| Musculoskeletal disorder index (+1SD) |  |  |  |  |  | 1.07 (1.01–1.13) |
