# Supplementary figures and images for: Explaining regional variation in elective hip and knee arthroplasties in Finland 2010 − 2017—a register-based cohort study
Source: BMC Health Serv Res. 2022 Jul 9;22:891. doi: 10.1186/s12913-022-08305-7 (PMC9270793; doi:10.1186/s12913-022-08305-7)

**THA, Model 4: 2010–2013**

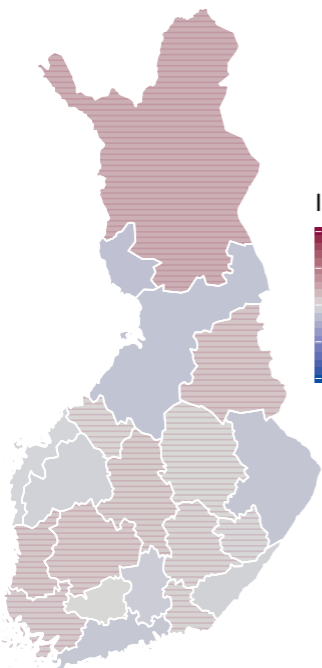

**THA, Model 5: 2010–2013**

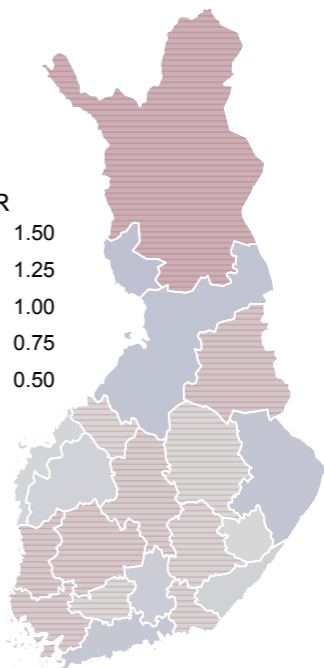

**THA, Model 4: 2014–2017**

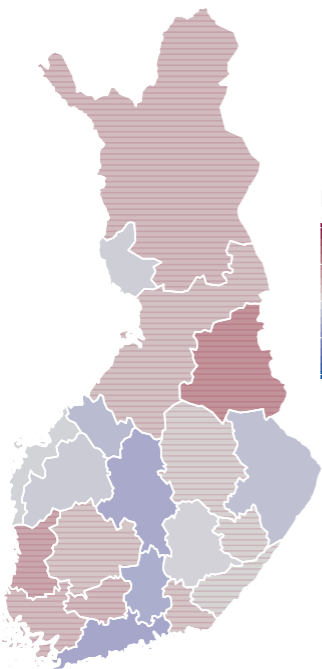

**THA, Model 5: 2014–2017**

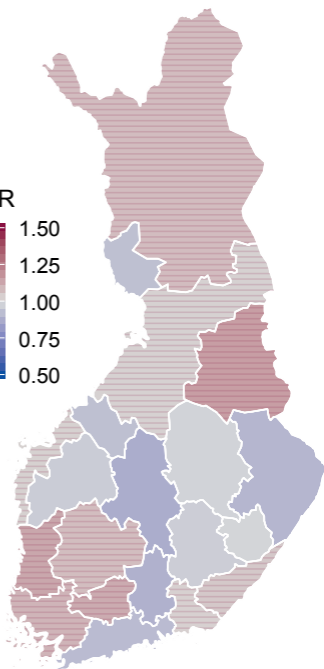

Supplement: Supplementary file 4 — Additional file 4. Hospital district variation of elective total hip (THA) arthroplasties controlling for age and gender only (Models 4 and 5) in 2010-2013 and 2014-2017. Striped areas have incidence rate rations (IRR) over the country average. [file 12913_2022_8305_MOESM4_ESM.pdf]

**TKA, Model 4: 2010–2013**

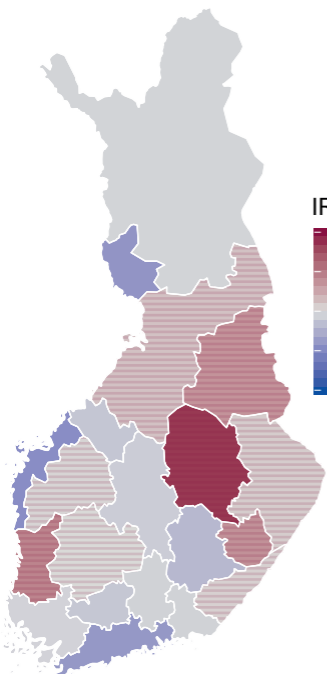

**TKA, Model 5: 2010–2013**

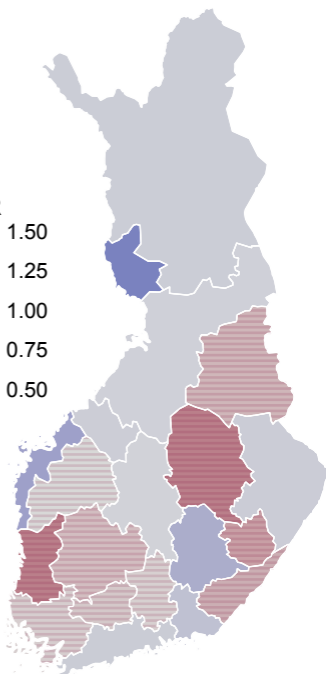

**TKA, Model 4: 2014–2017**

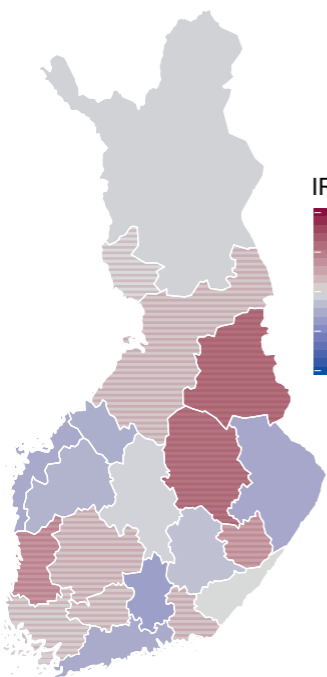

**TKA, Model 5: 2014–2017**

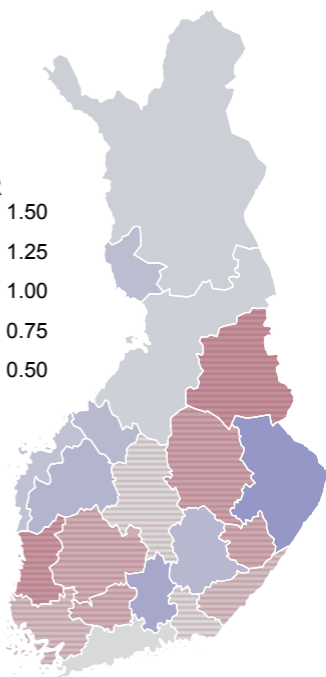

Supplement: Supplementary file 5 — Additional file 5. Hospital district variation of elective total knee (TKA) arthroplasties controlling for age and gender only (Models 4 and 5) in 2010-2013 and 2014-2017. Striped areas have incidence rate rations (IRR) over the country average. [file 12913_2022_8305_MOESM5_ESM.pdf]
